# Ambiguities of PGPR-Induced Plant Signaling and Stress Management

**DOI:** 10.3389/fmicb.2022.899563

**Published:** 2022-05-13

**Authors:** Siddhi Kashinath Jalmi, Alok Krishna Sinha

**Affiliations:** ^1^Department of Botany, Goa University, Goa, India; ^2^National Institute of Plant Genome Research, New Delhi, India

**Keywords:** microbial signaling, PGPR, plant signaling, hormones, stress response, plant development

## Abstract

The growth and stress responses developed by the plant in virtue of the action of PGPR are dictated by the changes in hormone levels and related signaling pathways. Each plant possesses its specific type of microbiota that is shaped by the composition of root exudates and the signal molecules produced by the plant and microbes. Plants convey signals through diverse and complex signaling pathways. The signaling pathways are also controlled by phytohormones wherein they regulate and coordinate various defense responses and developmental stages. On account of improved growth and stress tolerance provided by the PGPR to plants, there exist crosstalk of signaling events between phytohormones and other signaling molecules secreted by the plants and the PGPR. This review discusses some of the important aspects related to the ambiguities of signaling events occurring in plants, allowing the interaction of PGPR with plants and providing stress tolerance to the plant.

## Introduction

Plant growth-promoting rhizobacteria (PGPR) are known to positively stimulate the growth of plants by providing important nutrients and stress tolerance (Chen et al., 2006; Vansuyt et al., 2007; Tang et al., 2020). As biotic stress management, PGPR are known to suppress the growth of plant pathogenic microbes and are known to activate the plant's defense responses against the pathogens (Niu et al., 2011; Prasannakumar et al., 2015). Apart from biotic stresses, PGPR also have a positive role in conferring tolerance to the abiotic stressors like salinity, drought, and heavy metal stress (Gupta et al., 2002; Lim and Kim, 2013; Kim et al., 2014). The growth and stress response developed by the plant in virtue of the action of PGPR is dictated by the changes in hormone levels and related signaling pathways.

Each plant possesses its specific type of microbiota, and the structure of microbiota in the rhizosphere of the plant is shaped by the composition of root exudates secreted by the plant roots and the signal molecules produced by the plant and microbes (Chaparro et al., 2014; Nelson and Sadowsky, 2015; Zhang et al., 2017; Jalmi, 2020). With the help of these signaling molecules, different types of associations are made between plants' roots and microbes.

The plant consists of diverse signaling networks and pathways transmitting various extracellular and intracellular signals. Based on the stimuli or the signaling components, there are various signaling pathways like mitogen-activated protein kinase (MAPK) pathways and calcium-dependent protein kinase (CDPK) pathways working inside the plant (Hamel et al., 2006; Dodd et al., 2010a; Sinha et al., 2011; Bredow and Monaghan, 2019). These signaling pathways are very well-explored for their role in abiotic or biotic stress *per se*. They are also controlled by phytohormones wherein they regulate and coordinate various defense responses and developmental stages. Of these several signaling components, MAPKs are highly conserved signaling molecules consisting of three-tier components, MAPKKK, MAPKK, and MAPK, transmitting signals by phosphorelay mechanisms (Hamel et al., 2006). MAPKs are activated by specific cues; however, at times crosstalks occur in these pathways but often end up with specific responses (Sözen et al., 2020). MAPKs also are known to interact with other signaling molecules to transmit the stimuli, giving a specific response (Jalmi and Sinha, 2015; Jalmi et al., 2018). MAPKs are involved in the transmission of diverse stresses, both abiotic and biotic, as well as developmental cues (Sinha et al., 2011; Sethi et al., 2014; Jalmi and Sinha, 2016; Singh and Sinha, 2016; Verma et al., 2020). MAPKs play an important role in pathogen defense, wherein studies have been carried out in understanding upstream receptors/sensors and downstream targets of MAPKs (Meng and Zhang, 2013). In this regard, MAPKs such as MPK3, MPK4, MPK6, and MPK7 have been reported in imparting resistance to pathogenic microbes (Sheikh et al., 2013; Jalmi and Sinha, 2016). Under any stress, cells can quickly respond to the environment, which is due to the fluctuation of cytosolic calcium and the presence of Ca^2+^ sensing proteins like calmodulins and calcium-dependent protein kinases (CDPKs). In addition to MAPKs, CDPKs have also shown an important role in plant growth and development under abiotic stresses by modulating abscisic acid (ABA) signaling and regulating reactive oxygen species (ROS) accumulation (Asano et al., 2012). Calcium signaling also plays a crucial role in defense against plant pathogens wherein the CDPKs transmit the signals related to pathogens regulating the immune response of plants (Sardar et al., 2017; Bredow and Monaghan, 2019). However, the involvement of MAPKs and CDPKs in transmitting signals related to beneficial microbes has not been detailed completely.

Pathogen defense in the plant has been very well-evolved, with a powerful immune system consisting of two tiers of defense: PAMP-triggered immunity (PTI) and effector-triggered immunity (ETI). PTI is triggered by conserved microbial signatures called pathogen/microbe-associated molecular patterns (PAMP/MAMP), which are perceived by pattern recognition receptor (PRR) at the cell surface, leading to a basal level of the immune response. The second level of immunity, ETI, is developed against effector molecules injected by pathogens inside the plant to counteract the PTI. ETI is also called the R gene-dependent resistance or gene for gene resistance (Jones and Dangl, 2006). Defense responses in the plant are also controlled by plant hormones like salicylic acid (SA), jasmonic acid (JA), and ethylene (ET). These defense responses are systemic acquired resistance (SAR) and induced systemic resistance (ISR). SAR is induced by long-distance signaling by salicylic acid, resulting in the upregulation of defense response genes like pathogenesis-related genes (PR), whereas ISR is induced by jasmonic acid (JA) and ethylene in plants (Glazebrook, 2005; Jones and Dangl, 2006; Fu and Dong, 2013). Many PGPRs are studied to induce SAR by producing SA at the root interface, and some of the rhizobacteria induce SA independent ISR. *Pseudomonas* spp. and *Bacillus* spp. induce ISR and SAR in Arabidopsis and many other plants, thus providing resistance to a broad range of plant pathogens (Haas and Défago, 2005). Signaling events and the signaling components involved in plants during interaction with beneficial microbiota lack extensive information. Signaling components participating in symbiotic association with plants have been explored, but signaling phenomena occurring in non-symbiotic association need detailed understanding. Questions also arise about whether the same receptors and signaling components transmit the signals of beneficial microbes to plants as they do for pathogenic microbes. Also, if this is the fact, then how are the responses different for pathogenic and beneficial microbes? This review discusses some of the important aspects related to the crosstalk of signaling events occurring in plants, allowing the interaction of PGPR with plants, thus providing stress tolerance to the plant.

## Signaling Molecules at Plant Root Interface

**Figure 1:**
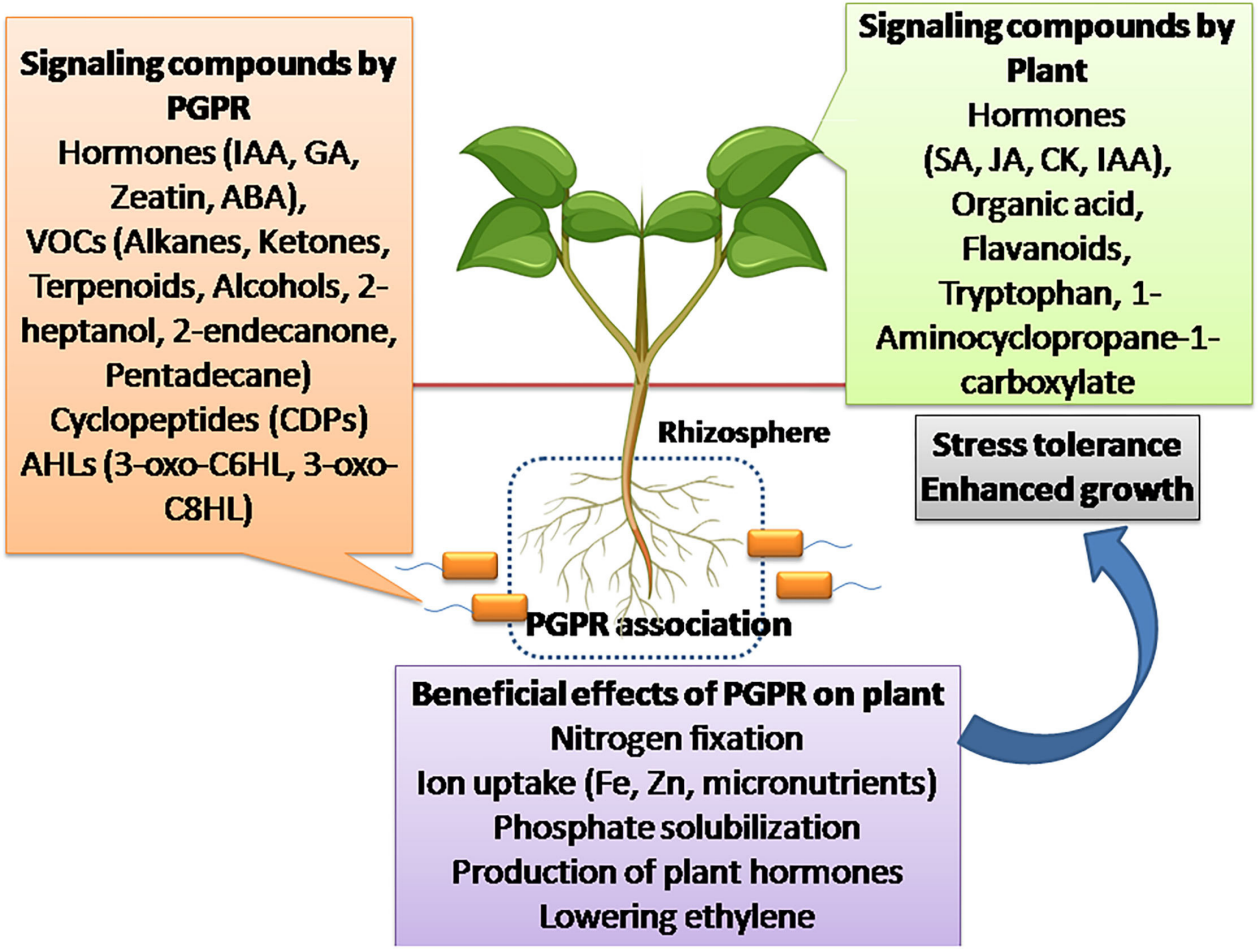
Signaling compounds produced by PGPR and plants for setting up the beneficial rhizospheric association. The compounds produced by PGPR include hormones (IAA, GA, Zeatin, ABA), ACC deaminase, VOCs (Alkanes, Ketones, Terpenoids, Alcohols, 2-heptanol, 2-endecanone, Pentadecane), cyclopeptides (CDPs), acyl homoserine lactones (AHLs) like 3-oxo-C6HL, 3-oxo-C8HL, which triggers plant signaling, helping in plant growth promotion and stress tolerance. Similarly, plants produce signaling molecules like plant growth hormones (SA, JA, CK, IAA) in response to PGPR, helping in their signaling and stress response. The associated PGPR improves plant growth by providing essential minerals through nitrogen fixation, ion uptake (Fe, Zn, micronutrients), and phosphate solubilization.

The most important molecular signals regulating plant growth and development are phytohormones. They have shown a profound role in various environmental stress and developmental signaling (Bedini et al., 2018). PGPR are known to produce a wide range of hormones acting as signal molecules in the rhizospheric region, thus allowing the interaction of PGPR with the plant roots and plant development. It has been studied that PGPR including *Bacillus amyloliquefacians, Pseudomonas fluorescence*, and *Bradyrhizobium japonicum* significantly produce plant growth hormones such as Indole-3-acetic acid (IAA), Gibberellic acid (GA), zeatin, ET, and ABA (Boiero et al., 2007) (Figure 1).

Indole-3-acetic acid produced by rhizobacteria is an essential hormone for root nodulation, vascular bundle formation, and cell division and differentiation. The biosynthesis of IAA by rhizobacteria is influenced by environmental factors and genetic factors (Spaepen et al., 2007). The majority of rhizobacteria studied are capable of producing IAA *via* indole-3-pyruvic acid and indole-3-acetic aldehyde pathway, some of which include: *Azorhizobium caulinodans, B. japonicum, Rhizobium japonicum, Rhizobium leguminosarum, Rhizobium meliloti, Rhizobium phaseoli, Rhizobium trifolii*, and *Sinorhizobium meliloti* (Yanni et al., 2001; Naidu et al., 2004; Boiero et al., 2007; Senthilkumar et al., 2009; Chi et al., 2010) (Table 1).

**Table 1:** Signaling compounds produced by PGPR.

**Signaling compounds**	**Role**	**PGPR**	**References**
**Hormones**			
Indole-3-acetic acid	Root nodulation, Vascular bundle formation Cell division and differentiation	*A. caulinodans, B. japonicum, R japonicum, R. leguminosarum, R. meliloti, R. phaseoli, R. trifolii, S. meliloti*	Yanni et al., 2001; Naidu et al., 2004; Boiero et al., 2007; Senthilkumar et al., 2009; Chi et al., 2010
Cytokinins	Seed germination, apical dominance, senescence	*B. subtilis, Paenibacilluspolymyxa*	Timmusk et al., 1999; García de Salamone et al., 2001; Liu et al., 2013
Gibberellins	Leaf expansion, Stem elongation,	*B. japonicum, B. pumilus, B. licheniformis, Rhizobium* species and *S. meliloti*	Katznelson and Cole, 1965; Arshad and Frankenberger, 1997; Boiero et al., 2007; Govindasamy et al., 2010
Ethylene	Fruit ripening and floral senescence	*B. subtilis, B. licheniformis, B. mycoides, Cryotococcusalbidus*	Fukuda et al., 1989; Govindasamy et al., 2010
ACC Deaminase	Lowers stress induce ethylene production by converting precursor ACC into ketobutyrate and NH_3_	*R. leguminosarum, R japonicum, R. gallicum, B. japonicum, B. eklani, S. meliloti, Variovorax* sp.	Duan et al., 2009; Onofre-Lemus et al., 2009; Gupta and Pandey, 2019; Bessadok et al., 2020
**(Volatile Organic Compound)VOCs**			
Alkanes, Ketones, Terpenoids, Alcohols, Sulfur compounds like 2-heptanol, 2-endecanone, and Pentadecane	Signals to cognate receptors for cell-cell communication and for communication with plants.	*B. subtilis, B. methylotrphicus, B. atrophaeus, Paenibacilluspolymyxa*	Zhang et al., 2007; Farag et al., 2013; Pérez-Flores et al., 2017; Ayaz et al., 2021
Cyclodipeptides (CDPs)	Lateral root development by acting as auxin like signal	*P. aeruginosa*	Ortiz-Castro et al., 2011
Lipo-chitooligosaccharide (LCO)	Nodulation, symbiotic association, lateral root formation through auxin homeostasis, activates plant immunity (ISR)	*Rhizobia sp., Bradirhizobium japonicum*, Arbuscular mycorrhizal fungi	Lian et al., 2002; Maillet et al., 2011; Oldroyd, 2013; Buendia et al., 2018

Cytokinins (CKs) are important hormones regulating seed germination, apical dominance, senescence, and interaction of the plant with microbes (Akhtar et al., 2020). Strains of rhizobacteria are also reported to efficiently produce cytokinin hormone, wherein they enhance the growth of shoot, lateral roots, and increase secretion of root exudates, further increasing the beneficial bacteria-plant interaction (García de Salamone et al., 2001; Senthilkumar et al., 2009; Liu et al., 2013; Asari et al., 2017) (Table 1).

Gibberellic acid is another phytohormone reported to be produced by PGPR which is responsible for leaf expansion and stem elongation. PGPR including *Azospirillum* spp., *Azotobacter* spp., *Bacillus* spp., and rhizobia produce GA (Dodd et al., 2010b). GA3 production by rhizobacteria *B. japonicum* was first reported by Katznelson and Cole (1965) and later reported by *Rhizobium* species and *S. meliloti* (Arshad and Frankenberger, 1997; Boiero et al., 2007). GA producing bacteria exhibit beneficial effects on plants promoting root and shoot growth and also improving seedling vigor (Yanni et al., 2001). Apart from GA3, *Rhizobium* and *B. japonicum* also produce a significant amount of ABA, which is an important hormone in providing tolerance to plants under drought stress (Boiero et al., 2007). Another study reports an increase in the level of ABA in plants when inoculated with *Azospirillum* spp. (Cohen et al., 2008) (Table 1).

The negative effects of ET hormone, i.e., inhibition of root elongation, transport of auxins, and leaf senescence were nullified by PGPR by producing enzyme 1-aminocyclopropane-1-carboxylate (ACC) deaminase controlling excessive ethylene production (Figure 1). Bacteria producing ACC deaminase are known to take up ACC produced by plants from the rhizosphere and convert it into ketobutyrate and NH_3_. The strains studied to be producers of ACC deaminase are *R. leguminosarum, R japonicum, Rhizobium gallicum, B. japonicum, Bradyrhizobium elkanii, S. meliloti*, and *Variovorax* sp. (Duan et al., 2009; Onofre-Lemus et al., 2009; Gupta and Pandey, 2019; Bessadok et al., 2020) (Table 1). Bacteria producing ACC deaminase enhanced root and shoot elongation, increased nodulation in legumes, and increased mineral uptake. This explains the fact that PGPR protects the plant from environmental stresses by lowering the levels of phytohormone ethylene. Other than the phytohormones, PGPR also produces volatile organic compounds (VOCs) which are used for the intra-bacterial communication. These VOCs include alkanes, ketones, terpenoids, alcohols, sulfur compounds, and so on, of which 2-heptanol, 2-endecanone, and pentadecane are studied to act as signals for communication with plants (Zhang et al., 2007; Farag et al., 2013; Pérez-Flores et al., 2017; Ayaz et al., 2021) (Figure 1). These aromatic lipophilic compounds act as signals to cognate receptors for cell-cell communication and are also used as signals for communication with plants (Table 1). Upon sensing these VOCs by the plant root system, it modulates the root architecture showing an ecological significance for strengthening of mutual interaction between plant roots and PGPR. A study in Arabidopsis reported that VOCs, such as aldehyde, 1-butanol, and ketones, secreted by *Bacillus* spp. caused modulation of root architecture and increased root biomass (Pérez-Flores et al., 2017). Apart from this, VOCs such as acetoin and 2,3-butanediol produced by *Bacillus* spp. had a stimulatory effect on plant growth. This stimulatory effect by VOCs could be due to the activation of hormonal signaling (Farag et al., 2013; Pérez-Flores et al., 2017). Yet another VOC, Butyrolactone, acts as an inducer of quorum sensing in bacteria, thereby stimulating more bacterial communications and plant root interactions (Polkade et al., 2016).

Besides signaling molecules produced by PGPR, the plant secretes low molecular weight, high molecular weight, volatile, and non-volatile compounds in the root exudates, which influences the interaction of various microbes in the rhizosphere. Studies have shown that signaling molecules such as coumarins, triterpenes, flavonoids, and benzoxazinoids allow the growth and inhibition of specific microbes in rhizospheric regions of plants (Hu et al., 2018; Cotton et al., 2019).

The roots of the majority of land plants produce a group of carotenoid-derived novel metabolites playing a role as phytohormones called Strigolactones (SLs). The SLs are involved in many of the important aspects of plant development earlier defined as ex-planta signaling molecules secreted by plants' roots inducing germination of parasitic plants. Later, they were found to help in establishing a symbiotic relationship between plants and arbuscular mycorrhizal fungi (Mori et al., 2016). The reports also suggest the role of SLs in regulating the positive interaction of *S. meliloti* with *Medicago sativa* (Soto et al., 2010).

It is now clear that the multitude of chemical compounds secreted by the PGPR and plant act as signaling molecules in influencing the rhizospheric associations and growth of the plant. With the help of metabolites produced, plants can influence microbiota and in turn microbiota influence the metabolites produced by the plants (Pang et al., 2021). Many studies have shown the positive effect of a secreted chemical compound on plant growth. Some studies have identified the PGPR organism and the related transcriptional activity without identifying secreted chemical compounds. The improvement in plant growth under environmental stress conditions imparted by PGPR is done by modulating intricate signaling and transcriptional activities, resulting in differential expression of genes. Studies show that PGPR ameliorate the stress conditions by modulating the expression of genes involved in hormonal biosynthesis, such as ACO and ACS genes (ethylene biosynthesis), MYC2 (Jasmonate), PR1 (SA), several genes encoding antioxidant enzymes (SOD, CAT, APX, GST), transcription factor NAC1, and so on (Tiwari et al., 2017). These studies need a correlation in order to understand the signaling networks and the mechanisms involved. Studies are required to identify the developmental signaling pathway in plants induced by signaling molecules secreted by the PGPR. This possibility is shown by a study of versatile signaling molecule cyclodipeptides (CDPs) involved in quorum sensing and PGPR interaction. One of the PGPR, *P. aeruginosa*, secreted CDPs that affected lateral root development in plants by acting as auxin-like signal and activating the auxin signaling pathway (Ortiz-Castro and López-Bucio, 2019) (Table 1).

## Convergence of Microbial and Plant Signaling Molecules

Lipo-chitooligosaccharide (LCO) also called Nod factors are signaling molecules, originally studied to be produced by *Rhizobia*, arbuscular, and ecto mycorrhizal fungi, helping in the nodulation process and establishing a symbiotic relationship with plants (Oldroyd and Downie, 2008; Maillet et al., 2011). Many other fungi belonging to Ascomycetes and Basidiomycetes are studied to be the producers of LCOs, wherein they act as signals regulating fungal growth and development (Rush et al., 2020). Apart from their important role in plant symbiotic association, they also have a direct impact on plant growth and development. They have been shown to increase the growth of the plant in stressed conditions and stimulate the lateral root formation (Zipfel and Oldroyd, 2017; Buendia et al., 2018). Studies show that the lateral root formation by the LCO is through the regulation of auxin homeostasis (Buendia et al., 2018) (Table 1). This opens an interesting avenue of the effects of microbial signaling molecules on hormone signaling in plants.

**Figure 2:**
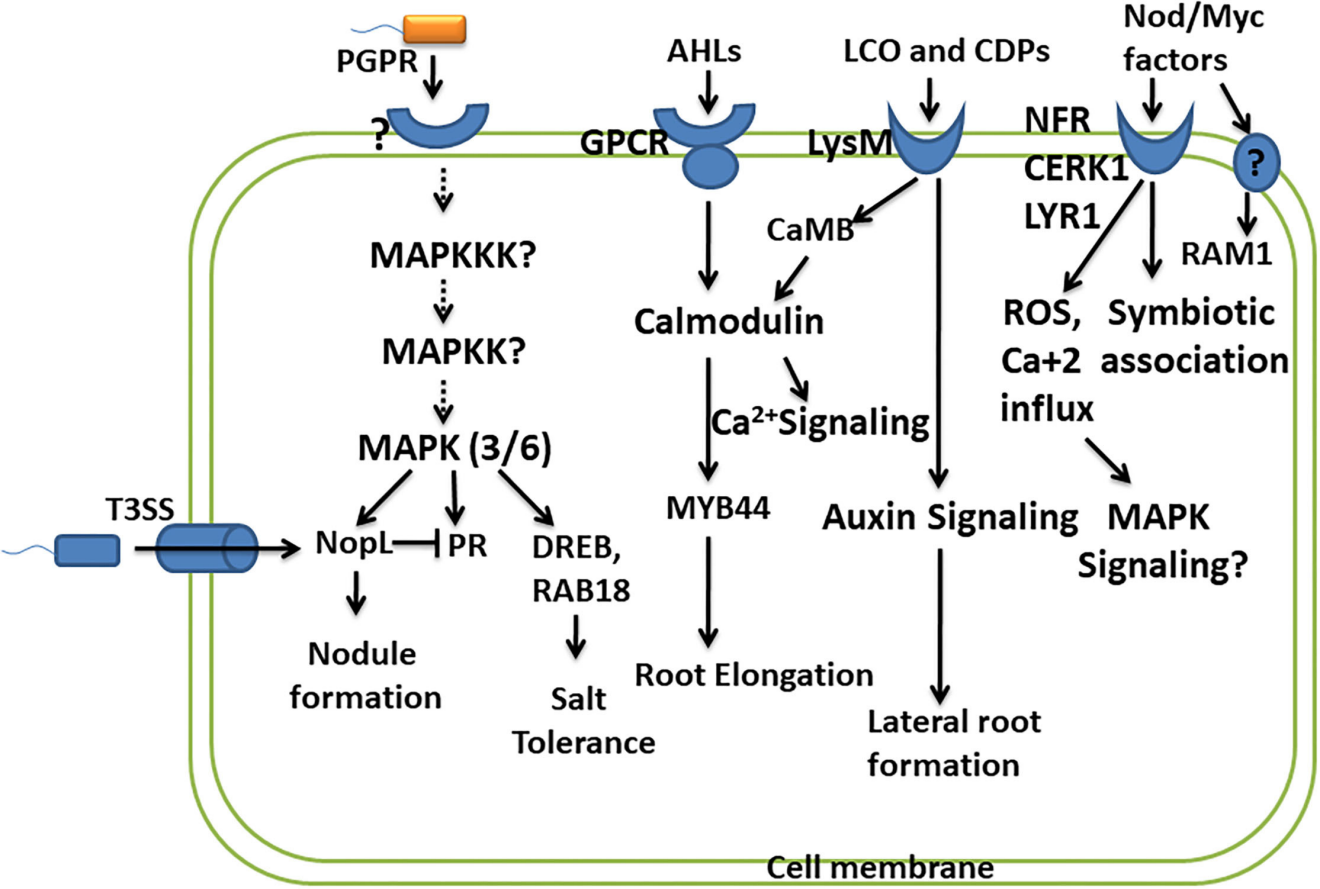
Convergence of PGPR and plant signaling pathways. This depicts the studies carried out in understanding the signaling molecules/pathways involved in perceiving PGPR and their response in promoting plant growth and development. The important signaling pathways involved are MAPK signaling, calcium signaling, and hormone signaling. However, many other components of these pathways and their role in response to PGPR remain to be studied.

Lipo-chitooligosaccharides produced by beneficial microbes are recognized by plant receptors, lysin motif receptor-like kinase (LysM-RLKs), containing oligosaccharide-binding LysM domain. It is presumed that binding of LCOs with LysM receptors activates the signaling pathway, leading to the oscillation of nuclear calcium concentration, leading to the formation of nodules, and promoting the symbiotic association. The symbiosis established by *Rhizobia* with leguminous plants is with the help of recognition of LCOs/Nod factors (NFs) by Nod factor receptors (NFRs) of leguminous plants. Two NFR receptors, NFR1 and NFR5, bind Nod factors directly (Oldroyd, 2013). A study reports that LYK10, LysM receptor-like kinases, show a high affinity for LCOs, and their interaction is essential for root nodule symbiosis (Girardin et al., 2019). Several LysM-RLKs have been identified in many plants like OsCERK1 in rice, SlLYK10 and SlLYK12 in tomato, and MtLYK9 in *Medicago truncatula*. OsCERK1 is known to perceive various other ligands like such as chitins, peptidoglycans, and chitiologosaccarides (COs), which shows the dual functions, i.e., in symbiosis as well as in plant defense (Ao et al., 2014). Here, the questions on how these interactions lead to two different responses, i.e., establishing a symbiotic association and initiating plant defense against pathogens, or of does pathogen utilize the same signaling pathways in establishing their own colonization arise. This fact has been explained very well in a study by Feng et al. (2019), wherein they suggest that AM fungi promote symbiotic signaling through a combination of LCOs and COs. Different CO molecules show different responses, and short-chain COs like CO4 and CO5 activate symbiotic signaling, whereas long-chain COs like CO8 induce immune signaling (Cao et al., 2017). However, Feng et al. (2019) suggested that there exists no difference in the perception and signaling of COs from CO4 to CO8. LCOs together with COs play a role in symbiosis and can suppress immunity signaling, thus promoting symbiotic outcomes. While the perception of only COs is required for immune responses, an important receptor kinase DMI2 plays an important role in the activation of symbiotic signaling in response to LCOs and COs but shows no role in plant immune signaling (Feng et al., 2019). LCO and CO perceived by DMI2 receptor kinase promote a symbiotic association possibly by creating nuclear-associated calcium oscillations. Perception of COs by CERK1 and LYK receptors activates plant defense by activating calcium influx across the plasma membrane, producing ROS, further activating MAPK signaling (Cao et al., 2017) (Figure 2). LCOs are also known to block the formation of ROS in response to pathogens without affecting plants' immunity (Rey et al., 2019). Accounting for the role of LCOs produced by both Rhizobia and AM fungi in symbiosis, the activation of common symbiotic genes and corresponding pathways by LCOs of both these microbes has been suggested by a previous study. However, it raises the question on specificity in activating these two pathways from different microbial symbionts. A study performed in the identification of specific components reported specific function of two important genes RAM1 (reduced arbuscular mycorrhization) and RAM2 in AM fungal symbiosis. RAM1 encodes the GRAS-transcription factor which regulates the expression of RAM2 encoding Glycerol-3-phosphate Acyl transferase (GPAT). Both RAM1 and RAM2 are specifically required for AM symbiosis and not in rhizobial symbiosis, of which RAM2 is very critical for induction of hyphopodia in AM fungi and also appressoria formation in *Phytophtora* infection (Gobbato et al., 2012, 2013) (Figure 2).

Like pathogenic bacteria which use type 3 secretion system (T3SS) to deliver virulence factors (T3 effector proteins), certain rhizobial strains also produce effector protein called nodulation outer protein L (NopL), affecting the nodule formation in symbiosis. NopL bypass the NFR for the formation of the nodule in the legume. NopL affects the process of nodule formation by interacting with plant signaling pathways. A study was performed to show that phosphorylation of NopL was inhibited by Ser/Thr kinase inhibitor and MAPKK inhibitor, suggesting NopL to be a substrate of MAPK (Bartsev et al., 2003; Ge et al., 2016) (Figure 2). NopL suppresses premature nodule senescence by interfering with MAPK signaling (Zhang et al., 2011). NopL also shows indirect interaction with the MAPK pathway by blocking the transcription of PR proteins like glucanase and chitinase, both of which are regulated by the MAPK pathway (Bartsev et al., 2004; Ge et al., 2016). These studies show the importance of the MAPK pathway as a convergent point involved in transmitting the signals for establishing symbiosis (Figure 2). Another study revealed that Bel 2-5 effectors produced by Rhizobia hijack the legume nodulation signaling by Nod factors and NFRs. Bel 2-5 resembles the XopD effector produced by plant pathogen *Xanthomonas campestris*. The induction of nodulation by Bel 2-5 effector is through the expression of cytokinin-related genes involved in nodule organogenesis and repressing ethylene and defense-related genes (Ratu et al., 2021).

As described earlier, LCOs influence the plant growth promotional activities by altering the plant hormone homeostasis, leading to improved photosynthesis and enhanced resistance to the environmental stresses. For LCOs to exert their effect, plants perceive these signaling molecules through the plant receptors. These receptors are LysM and belong to the lysine motif containing receptor-like kinase family (Liang et al., 2014). They are present in all microbial domains except Archea and are studied to interact with and respond to microbial-associated molecular pattern (MAMPs) (Gust et al., 2012). Intense studies determining the signal transduction by the perception of LCOs by LysM receptors are missing. However, a transcriptome analysis in response to LCO in plants suggested a strong induction of genes encoding calmodulin-binding protein CaMB (Zeng et al., 2015) (Fig. 2). Calmodulin binding proteins are major calcium sensors and regulate broad spectrum of target proteins. Induction of CaMB by LCO shows its possible involvement in calcium signaling in plants. Signaling of AHLs like 3-oxo-C6-HL and 3-oxo-C8-HL is mediated through G-protein coupled receptors in plants, wherein they exert unknown effect on the activity of calmodulin and transcription factor MYB44 involved in root elongation response (Zhao et al., 2015, 2016) (Figure 2). Another R2R3 MYB-like transcription factor MYB72 exhibits its role in early signaling of rhizobacteria-mediated jasmonate/ethylene-dependent ISR (Van der Ent et al., 2008) (Figure 2).

A few studies have reported the interlinking of signaling components in stress tolerance mediated by PGPR. A very important CBL-interacting protein kinase CIPK24 involved in calcium signaling is required for the proper functioning of K^+^ transporter by phosphorylating SOS1 (Na^+^/H^+^ antiporter) (Halfter et al., 2000). Also, another CBL-interacting protein kinase, CIPK12, imparts tolerance to salt stress in Arabidopsis. This suggests the involvement of calcium signaling components in the regulation of ion transporters and salt tolerance. As PGPR provides salt tolerance by various mechanisms by controlling ion uptake and homeostasis, this could be due to the effect of PGPR on such signaling pathways which are known to be involved in providing salt tolerance.

The stress tolerance is ameliorated by PGPR by modulating several genes related to hormonal biosynthesis and defense response, which propose an interlink between PGPR and signaling pathways. *Enterobacter* species ameliorated the salt stress by inducing salt stress-responsive genes such as DRE-binding protein (DREB), late embryogenesis abundant (RAB18), and also the important players of MAPK signaling cascade MPK3 and MPK6, thus increasing the ROS scavenging activity (Ilangumaran and Smith, 2017) (Figure 2). These reports suggest connecting bridges between rhizobacteria perception by signaling pathways and its subsequent transmission for improving the health of the plants in environmental stresses. However, information is still required to fill the necessary gaps.

## Plant Stress Tolerance Linked to PGPR

Plants being sessile are exposed to a wide range of environmental stresses generally grouped into abiotic and biotic stresses. Work is being carried out to study different mechanisms of stress tolerance. Plant breeders use the long, capital-intensive breeding method to obtain tolerant plant varieties. However, beneficial PGPR plays an important role in inducing stress tolerance, thus giving importance to stress management. PGPR are known to produce an array of compounds that act as signaling molecules activating and altering several cellular processes, thus conferring stress tolerance to plants. Here, with the help of some reports, we will discuss how stress tolerance is obtained in PGPR-plant interaction.

## Abiotic Stress Management

One of the most important PGPR, *Pseudomonas putida* strain KT 2440, has agronomical importance and has been studied to impart a wide range of tolerance to abiotic as well as toward certain pathogens. Being salt-tolerant bacteria, it improves plant growth and seed germination under saline conditions. The salinity tolerance exhibited by *P. putida* KT2440 is due to the biosynthesis of lipopolysaccharides (LPS) by the enzyme phosphoethanolamine-lipid-A transferase (EptA) (Costa-Gutierrez et al., 2020). PGPR also provide salt stress tolerance by producing exopolysaccharide (EPS) that covers the root surface and prevents the influx of Na^+^ ions into the root cells. A study reported two PGPR species, *Aeromonas hydrophilla* and *Bacillus sp.*, that colonies the wheat roots and produces EPS, thus trapping Na^+^ and making it unavailable for the plant (Ashraf et al., 2004) (Table 2). However, the biosynthesis of EPS is regulated by the regulation of the gene encoding enzyme EptA. The *eptA* gene is induced by two components signaling involving PmrA and PmrB, with them being the major regulators. In this regulatory signaling component, PmrA is a cytoplasmic response regulator and PmrB is a membrane-bound sensor kinase (Chen and Groisman, 2013). PmrB kinase is studied to be activated by various signals like high metal ions, low pH, and vanadate, which phosphorylates PmrA and causes the activation of the *eptA* gene, further causing EPS production (Gunn, 2008). In response to various environmental stresses, the bacteria synthesizes EPS, which gives protection toward stresses. The major regulatory components, PmrA/PmrB and EptA enzymes, could be under the regulation of various other signaling molecules that could be explored in depth.

**Table 2:** Mechanism of stress tolerance by PGPR.

**PGPR**	**Stress tolerance**	**Mechanism of stress tolerance**	**References**
*Pseudomonas putida* KT2440	Improved plant growth and seed germination in salt stress	Biosynthesis of lipopolysaccarides (LPS) by the enzyme phosphoethanolamine-lipid-A transferase (EptA)	Costa-Gutierrez et al., 2020
*Aeromonas hydrophilla* and *Bacillus* species	Salt stress tolerance	Produces extrapolysaccarides (EPS), trapping Na^+^ thus making it unavailable for the plant	Ashraf et al., 2004
*Bacillus subtilis* GB03	Salt stress tolerance	Downregulates high-affinity K^+^ transporter (HKT2) expression and upregulates HKT1 and SOS1, reducing the Na^+^ uptake and accelerating the transport of Na^+^ from leaves to roots	Zhang et al., 2008; Niu et al., 2016; Qin and Huang, 2020
*Azotobacter* strains	Salt stress tolerance	Enhances the uptake of K^+^ leading to accumulation of proline, polyphenols	Rojas-Tapias et al., 2012
*Variovorax paradoxus* 5C-2,	Salt stress tolerance, increased photosynthesis and biomass under salt stress	Produce enzyme 1-aminocyclopropane-1-carboxylate (ACC) deaminase, limits Na^+^ accumulation	Wang et al., 2016
*Enterobacter* species	Salt stress tolerance	Increases the activity of antioxidant enzymes (SOD, APX, CAT) and upregulates ROS pathway genes	Habib et al., 2016
*Bacillus spizizenii* FMH45	Salt stress tolerance	Production of siderophores, IAA, hydrolytic enzymes and phosphate solubilization, improved chlorophyll content, membrane integrity, and phenol peroxidase levels	Masmoudi et al., 2021
*P. putida* (H-2-3), *P. putida* MTCC5279	Improve salt and drought tolerance	Reprogramming the chlorophyll content, stress hormones like salicylic acid and abscisic acid, expression of antioxidants; modulating osmolyte accumulation, ROS scavenging ability, and membrane integrity	Kang et al., 2014; Tiwari et al., 2016
*Pseudomonas fluorescence* REN1	Improved the growth in flooded conditions by increasing root elongation, submergence stress tolerance	Produce ACC deaminase	Etesami et al., 2014
*Serratia nematodiphila*	Cold stress tolerance	Production of Gibberellin, ABA and lowering the levels of SA and JA	Asaf et al., 2017
*Burkholderiaphytofirmans*PsJN and *Pseudomonas* species	Provided cold stress tolerance	Modulation of carbohydrate metabolism and increased expression of cold acclimation genes and antioxidant activity	Fernandez et al., 2012; Subramanian et al., 2015
*Bacillus pumilus, B. sutilis, Pseudomonas fluorescence*, and *P. putida*	Defense against plant pathogens	Cell wall modification through lignin deposition; induce ISR, improve photosynthetic performance, photochemical parameters, and gas exchange	Anderson and Guerra, 1985; García-Gutiérrez et al., 2013; Samaniego-Gámez et al., 2021
*Bacillus amyloliquefaciens* (SN13)	Biotic stress tolerance	Modulation of phytohormone signaling, producing secondary metabolites, osmoprotectants, and scavenging ROS	Chen et al., 2016; Srivastava et al., 2016; Tiwari et al., 2017
*P. fluorescence* CHA0	Biotic stress tolerance	Accumulation of SA and induction of PR protein	Maurhofer et al., 1994

Besides producing EPS and protecting plant roots against adverse effects of stresses, PGPR also use other mechanisms for providing tolerance. In plants, high-affinity K^+^ transporter (HKT) controls the Na^+^ import and overexpression of this transporter fails to impart salt tolerance, making plants more susceptible (Zhang et al., 2008). It is studied that PGPR *Bacillus subtilis* GB03 inoculation downregulates the HKT expression, thereby reducing the Na^+^ uptake and providing salt tolerance to plants (Zhang et al., 2008). More specifically, it is reported that *Bacillus subtilis* GB03 downregulates HKT2 but upregulates HKT1 and SOS1, thereby imparting tolerance (Niu et al., 2016). Also, further restricting the uptake of Na^+^ by the roots causes induction of HKT, causing transport of Na^+^ from the leaves to the roots, thereby reducing the salt stress effects (Qin and Huang, 2020). Apart from restricting the uptake of Na^+^, some PGPR-like *Azotobacter* strains enhance the uptake of K^+^ that leads to the accumulation of proline, polyphenols, and so on (Rojas-Tapias et al., 2012) (Table 2). To understand this mechanism of salt tolerance in the presence of PGPR, it will be important to understand the signaling components regulating the HKT and other important transporters. A MAPK signaling cascade studied to provide salt tolerance by regulating proline levels includes MKK3-MPK6-MYC2, which points out one possible signaling pathway that could regulate this process (Verma et al., 2020).

Yet another mechanism for salt tolerance is the production of ACC deaminase. Rhizospheric bacteria, *Variovorax paradoxus* 5C-2, has been studied to produce enzyme 1-aminocyclopropane-1-carboxylate deaminase, which promotes the growth of plants by lowering the ABA levels in plants, limiting Na^+^ accumulation under salt stress. This effect was studied in the *Pisum sativum* plant and was studied to have increased photosynthesis and biomass under salt stress when treated with *V. paradoxus* (Wang et al., 2016). It has been studied that the ACC deaminase produced by PGPR lowers the level of ABA, whereas other growth hormones produced by PGPR promote plant growth and impart salt tolerance by regulating the production of secondary metabolites (Kang et al., 2019). Another ACC deaminase producing rhizobacteria of *Enterobacter* species enhances salt tolerance by increasing the activity of antioxidant enzymes, such as SOD, APX, CAT, and upregulation of ROS pathway genes (Habib et al., 2016) (Table 2). ROS are one of the important messengers activating many cellular signaling pathways such as MAPK signaling pathways, involved in combating environmental stresses (Jalmi and Sinha, 2015). This could be yet another point of convergence of bacterial and plant signaling which needs further exploration.

Hormones play a very important role in combating stress. Several hormones are already reported to have a major role in stress tolerance (Ryu and Cho, 2015). PGPR are very well-discussed in hormone production. Production of IAA, cytokinin, gibberellins, and ABA by PGPR was found to impart salt tolerance in many plant species (Dodd et al., 2010b). According to the reports, around 80% of rhizobacterial isolates possess the ability to produce IAA as one of the secondary metabolites. The IAA secreted by bacteria acts as a signaling molecule in plants and alters many of the plant developmental processes, including the endogenous plant IAA levels (Spaepen et al., 2007). Cellular auxin homeostasis becomes necessary to have proper morphogenesis and stress responses because of overproduction or uptake of auxin developmental defects and impaired stress responses in plants. In this process, many of the plant signalings like MAPK and calcium signaling are involved in maintaining the auxin levels. In turn, auxin mediates the induction of these signaling pathways, and they further alter many cellular processes during the responses to a wide range of environmental stresses (Mockaitis and Howell, 2000; Vanneste and Friml, 2013). A recent study suggests the role of a halotolerant bacteria, *Bacillus spizizenii* FMH45, in the alleviation of salt stress in tomato plants. This bacterium is studied to be a potential producer of IAA, siderophores, and hydrolytic enzymes and is capable of phosphate solubilization, thus improving chlorophyll content, membrane integrity, and phenol peroxidase levels in imparting salt tolerance to plant (Masmoudi et al., 2021).

Apart from IAA production, rhizobacteria are also known to be producers of other stress hormones. The strain of *P. putida* (H-2-3) was reported to improve salt and drought tolerance by reprogramming the biosynthesis of stress hormones, such as salicylic acid and abscisic acid, chlorophyll content, and expression of antioxidants (Kang et al., 2014). These ABA and SA signaling pathways are identified as central regulators of abiotic and biotic stresses, wherein they induce various other plant signaling pathways like MAPK and calcium signaling and alter the gene expression of many stress response genes (Danquah et al., 2014; Edel and Kudla, 2016).

A growing body of studies of different strains of *P. putida* reveals its role in imparting tolerance to abiotic stresses other than salinity. A strain MTCC5279 mitigated drought stress in *Cicer arietinum* by modulating osmolyte accumulation, ROS scavenging ability, and membrane integrity. Also, modulation of gene expression involved in hormone biosynthesis and antioxidant enzyme suggested the involvement of stress hormone and antioxidant enzyme in giving stress tolerance (Tiwari et al., 2016) (Table 2). Apart from salinity and drought tolerance, PGPR also provide tolerance to submergence stress. A study demonstrated that *Pseudomonas fluorescence* REN1, when inoculated on rice seedlings, improved the growth in flooded conditions by increasing root elongation, possibly by producing ACC deaminase (Etesami et al., 2014). PGPR are also equally known to provide tolerance to heat and cold stress. Gibberellin-producing PGPR *Serratia nematodiphila* helped in better growth of the plant under low temperatures. This effect was due to the production of hormones GA and ABA and the lowering of the levels of SA and JA (Asaf et al., 2017). Similarly, *Burkholderia phytofirmans* PsJN and *Pseudomonas* species provided cold stress tolerance by modulating carbohydrate metabolism and increasing the expression of cold acclimation genes and antioxidant activity (Fernandez et al., 2012; Subramanian et al., 2015) (Table 2).

## Biotic Stress Management

One of the mechanisms shown by PGPR in promoting plant growth is through suppressing plant diseases; this can occur through microbial antagonism or by inducing resistance in plants against pathogenic microbes. Resistance conferred is due to the priming of effective resistant mechanisms possibly by ISR and SAR, both of which represent basal resistance depending on signaling hormones jasmonic acid and salicylic acid (Van Loon et al., 1998). The phenomenon of induction of ISR by PGPR was first reported by Van Peer et al. (1991) and Wei et al. (1991), wherein some of the rhizobacterial strains provided resistance against pathogens *Fusarium oxysporum* and *Colletotrichum orbiculare*. The mechanisms of resistance shown by the PGPR against plant pathogens are the production of hydrolytic enzymes, siderophores, antibiotics, and regulation of plant ethylene levels (Glick and Bashan, 1997; Neeraja et al., 2010). The most important PGPR studied to induce ISR belong to *Pseudomonas, Serratia*, and *Bacillusspp* (Van Wees et al., 2008).

It has been difficult to point out and differentiate the ISR induced by beneficial microbes and pathogenic microbes since ISR can be induced by several beneficial and pathogenic microorganisms and also other stresses activating the same type of response. Over past decades, several bacterial components have been identified to trigger ISR, which include lipopolysaccharides in cell envelop, flagella, AHLs, exopolysaccarides, secreted metabolites, and quorum sensing molecules (Ryu et al., 2004; Ortmann et al., 2006). Defense mechanisms shown by the plant in response to non-pathogenic microbes are callose deposition, production of phytoalexin, and pathogenic-related proteins (PR proteins), similar to responses shown by pathogenic infection (Hammond-Kosack and Jones, 1996; Raj et al., 2012). Cell wall modification through lignin deposition is one of the mechanisms of defense response shown by PGPR. This response was observed in *Bacillus pumilus, B. subtilis, Pseudomonas fluorescence*, and *P. putida* (Anderson and Guerra, 1985; García-Gutiérrez et al., 2013). *Bacillus amyloliquefaciens* (SN13) has been reported to act as a biocontrol agent apart from its beneficial role in imparting salt tolerance. This bacterium exerts its effect by modulating phytohormone signaling, producing secondary metabolites, osmoprotectants, and scavenging ROS (Chen et al., 2016; Srivastava et al., 2016; Tiwari et al., 2017) (Table 2). Some isolates of *Bacillus* spp. improved plant growth and provided resistance by inducing ISR against PepGMV, wherein there was improved photosynthetic performance, photochemical parameters, and gas exchange (Samaniego-Gámez et al., 2021) (Table 2).

Induced systemic resistance mediated by PGPR resembles the SAR induced by the pathogen, in that, in both responses, plants get more resistant to pathogenic microbes and one strain induces broad-spectrum resistance toward several pathogens in the same plant. In SAR, the SA produced activates specific sets of defense-related PR proteins that enhance the defensive capacity of the plant, which is normally not observed in ISR induced by the pathogen (Van Loon, 2007). Non-pathogenic microbes are generally studied to induce ISR consisting of jasmonate, and ethylene response and pathogenic microbes are known to induce SAR response (Van Loon et al., 1998). However, a study by Maurhofer et al. (1994) suggested the accumulation of SA-induced PR protein upon inoculation with PGPR *P. fluorescence* CHA0 (Table 2). The ISR by non-pathogenic microbes is dependent on NPR1 protein like that of pathogen-induced SAR and unlike that of ISR (Ryu et al., 2004). NPR1 along with the transcription factor TGA is a master regulator protein required for the signal transmission of SA in SAR (Shah et al., 1997). The NPR1 and TGA regulators are known to be regulated by members of the MAPK signaling pathway (Ekengren et al., 2003). The role of MAPK signaling and calcium signaling in plants is very well-explored in activating the SA- and JA-mediated SAR and ISR resistance (Tena et al., 2011). Many of the MAPK members have been studied to be activators of PR genes leading to the development of induced disease resistance (Meng and Zhang, 2013). More and more signaling pathways working in PGPR mediated ISR needs to be explored further. Also, the specificity in receptors and signaling molecules that differentiates pathogenic microbes and PGPR responses remains an unrevealed area.

These reports suggest that signaling pathways that are activated by the plant in response to pathogenic microbes and PGPR overlap with each other and indicate that they are regulated and balanced. The signaling pathways are controlled by hormones, and they do show interactions with each other. Over past years, several regulatory molecules have been identified in the interplay between pathways leading to SAR and ISR; however, their exact role remains to be elucidated.

## Conclusion and Perspective

Surveying the eco-friendly plant growth-promoting activities of PGPR, these bacteria can be exploited as efficient biotechnological tools in improving plant growth and crop yield in a stressful environment Qin et al., 2016. These bacteria are acting as promising fertilizers in agriculture and even as potential cleaners of the toxic environment by the process of remediation. The use of biocontrol PGPR replacing chemical pesticides is considered to be a better option for sustainable crop production (Bhardwaj et al., 2014). Some of the techniques used for sustainable agriculture are the use of these microbes or genetically engineered microbes as biofertilizers to enhance plant growth. PGPR-based biofertilizers are much safer for human health and the environment and are more readily degraded in soil. Biofertilizers with a single strain of PGPR or a consortium of multiple strains have been successfully used on various crop plants against various plant pathogens in multiple modes of application. For the commercialization of PGPR, it has to be passed from different stages, starting from its production in the laboratory to the farmer. Factors to take into consideration for the selection of strain or consortia and its commercialization are the type of crop, ecological zones, climatic, and soil conditions. All these factors affect the performance and effectiveness of the PGPR (Compant et al., 2010). Due to the variability and inconsistency observed in the performance of PGPR in the laboratory and in the field, efforts have been made to overcome this drawback by use of biotechnological techniques such as micro-encapsulation and nano-encapsulation. The stages involved in commercializing the PGPR strain as a biofertilizer includesL: field survey, isolation and characterization of PGPR based on soil properties and type of crop, method of inoculation and the carrier material to be used along, and extensive pot trials and field trials (Tabassum et al., 2017). The promising strains of PGPR used commercially as biofertilizers are the strains of *Pseudomonas, Paenibacillus, Bacillus, Brevibacillus, Achromobacter, Trichoderma*, and *Enterobacter* for various crop species like rice, soybean, pearl millet, peas, tomato, peanut, cotton, potato, and so on (Tabassum et al., 2017).

Plant growth-promoting rhizobacteria are known to show various direct and indirect mechanisms of enhancing plant growth and development. Their interaction with plants and the type of microbes present in the rhizospheric region of plant roots are specified by the chemical compounds acting as signaling molecules, secreted by both microbes and plant roots. PGPR produce diverse sets of signaling molecules at the plant root interface, which is essential to set up beneficial associations. These signaling molecules are perceived, and signals are transmitted by plant signaling pathways, to stimulate and alter genetic and molecular mechanisms leading to beneficial responses. The responses, such as enhanced plant growth and stress tolerance, also suggest the regulation of hormonal pathways. Plant signaling has been very well-studied in different environmental stresses and is known to play an important role in combating plant stress. The signaling pathways working in the perception and transmission of signals from pathogens are well-known; however, information regarding the perception and transmission of signals for beneficial microbes is limited. Extensive studies revealing the mechanisms by which plant determines the interaction with specific microbes and the factors involved in their coexistence is needed. Further, studies determining exudates dependent changes in plant microbiota and the screening of interaction of plant signaling molecules with microbial components will help provide further insights. This review is an attempt to focus on plant signaling interplay in PGPR association so that we can further explore the mechanisms and interlinks of signaling networks. Understanding the action of PGPR signaling molecules will allow more effective application of PGPR in the field.

## Author Contributions

SJ conceptualized the idea, wrote the manuscript, and prepared the illustrations. AS wrote and edited the manuscript. Both authors contributed to the article and approved the submitted version.

## Funding

The SJ Lab was supported by start-up grant (UGC-BSR) from University Grant Commission, Govt. of India, and DST-SERB start-up grant (File no. SRG/2021/001830-G) Govt. of India.

## Conflict of Interest

The authors declare that the research was conducted in the absence of any commercial or financial relationships that could be construed as a potential conflict of interest.

## Publisher's Note

All claims expressed in this article are solely those of the authors and do not necessarily represent those of their affiliated organizations, or those of the publisher, the editors and the reviewers. Any product that may be evaluated in this article, or claim that may be made by its manufacturer, is not guaranteed or endorsed by the publisher.
